# Evaluation and comparison of adaptive immunity through analyzing the diversities and clonalities of T-cell receptor repertoires in the peripheral blood

**DOI:** 10.3389/fimmu.2022.916430

**Published:** 2022-09-08

**Authors:** Yue Zhuo, Xin Yang, Ping Shuai, Liangliang Yang, Xueping Wen, Xuemei Zhong, Shihan Yang, Shaoxian Xu, Yuping Liu, Zhixin Zhang

**Affiliations:** ^1^ Department of Health Management & Institute of Health Management, Sichuan Provincial People’s Hospital, University of Electronic Science and Technology of China, Chengdu, China; ^2^ Department of Technology, Chengdu ExAb Biotechnology, LTD, Chengdu, China

**Keywords:** TCR, TCR repertoire, TCR diversity, aging, cancer

## Abstract

The adaptive immune system plays an important role in defending against different kinds of diseases, including infection and cancer. There has been a longtime need for a simple method to quantitatively evaluate the potency of adaptive immunity in our bodies. The tremendously diversified T-cell receptor (TCR) repertoires are the foundation of the adaptive immune system. In this study, we analyzed the expressed TCRβ repertoires in the peripheral blood of 582 healthy donors and 60 cancer patients. The TCR repertoire in each individual is different, with different usages of TCR Vβ and Jβ genes. Importantly, the TCR diversity and clonality change along with age and disease situation. Most elder individuals and cancer patients have elevated numbers of large TCRβ clones and reduced numbers of shared common clones, and thus, they have very low TCR diversity index (D_50_) values. These results reveal the alteration of the expressed TCRβ repertoire with aging and oncogenesis, and thus, we hypothesize that the TCR diversity and clonality in the peripheral blood might be used to evaluate and compare the adaptive immunities among different individuals in clinical practice.

## Introduction

The adaptive immune system is important to combat different diseases, including defending against various bacterial or viral infections and fighting cancer (1–3). There has been a long time request in clinical practice for a simple and quick method to quantitatively evaluate the potency of immunity, for example, to monitor immediate effects during immune cell therapy or to predict treatment effects in cancer patients. Different methods, including white blood cell counts, serum antibody levels, and fluorescence-activated cell sorter (FACS) analyses of lymphocyte subpopulation, have been applied to evaluate the potency of the adaptive immunity of the human body. However, these methods can only identify individuals with severe changes or defects in the immune system; they can neither distinguish healthy donors from cancer patients nor tell the differences among healthy people.

The adaptive immune system contains B and T lymphocytes. B and T cells express antigen receptors on their cell surface to recognize different antigens (1, 3). The B-cell receptor (BCR) is composed of two identical heavy chains and two identical light chains, while the T-cell receptor (TCR) is a heterodimer (1). To recognize almost unlimited types of antigens from invading viruses and microbes to endogenous malignant cells and foreign tissue grafts, BCR and TCR have developed vast diversified variable regions in the N-terminals of their polypeptide chains (1, 4–6). The diversities of BCR and TCR are generated through somatic DNA rearrangement during the early stages of lymphocyte development (4, 5, 7). Theoretically, there could be more than 1×10^18^ possible BCR types and 1×10^13^ possible TCR types (6, 8–11).

The tremendously diversified repertoires of BCR and TCR are the foundation of an adaptive immune system (3, 10, 12). BCR/TCR repertoire analyses have a lengthy research history and interest in understanding the changes in the immune system during development, aging, and diseases (6, 13–18). Accumulating studies showed that the TCR repertoire diversities declined with age (19–21). This is consistent with the common recognition that the adaptive immunity of elders is significantly impaired (22). It has also been reported that the diversity indexes of TCR repertoire are correlated to the prognosis of certain diseases, including cancer and viral infection (23, 24). Biased TCR repertoires were observed in the peripheral blood and in tumor-infiltrating lymphocytes of cancer patients. (25). However, most of these studies only analyzed a limited number of samples.

In this study, after analyzing the expressed TCRβ repertoires in the peripheral blood of 582 healthy donors, 60 cancer patients, and 12 cord blood samples, we revealed an association between age, cancer, and other factors with the expressed TCRβ repertoire. The results show that the TCRβ repertoire of each individual has different signatures. The diversity index (D_50_) values of TCRβ repertoire are reduced significantly in old people or cancer patients. Such changes are correlated with elevated numbers of large TCRβ clones and reduced numbers of shared common clones. We propose the hypothesis that the diversity index of the TCRβ repertoire in the peripheral blood might be used to evaluate and compare the adaptive immunities among different individuals in clinical practice in the future.

## Materials and methods

### Ethics statement and sample collection

The experimental design and participant recruitments were approved by the Medical Ethics Committee of Sichuan Provincial People’s Hospital (Ethics Approval for Research: 2020 #351). Individuals with acute infection, severe allergy, active autoimmune disease, immune deficiency, or who have had bone marrow transplantation were excluded. Cancer patients with ongoing chemotherapy or radiotherapy were also excluded. All enrolled subjects signed an informed consent form prior to the sample collection. All experiments were performed according to the relevant guidelines and regulations. Every participant donated approximately 2 ml of peripheral blood, which was collected *via* venipuncture with lavender top tubes using ethylene diamine tetraacetic acid (EDTA) as an anticoagulant.

### Lymphocyte separation

The collected peripheral blood sample was diluted to a total volume of 25 ml with normal saline (sterile 0.9% sodium chloride solution) and gently mixed well. The diluted blood was then slowly added on top of 25 ml of lymphocyte separation medium (Tian Jin Hao Yang Biological Manufacture Co., Ltd, Tianjin, China CAT# LTS1077) in a 50-ml conical tube. It was essential not to disrupt the interface between the blood and the medium. The loaded tube was centrifuged for 30 min at 500*g* in a swing-out rotor (Thermol Sorvall ST 40R with TX-1000 rotor Thermo Fisher Scientific, Waltham, MA, USA) with acceleration and deceleration speed setting at level 2. The supernatant was removed carefully, and the white cloudy content near the middle of the tube was collected and transferred into a new 50-ml conical tube. The tube was filled with normal saline and centrifuged for 8 min at 300*g*. The supernatant was discarded, and the pellet was resuspended with 20 ml of normal saline, and then centrifuged again for 5 min at 400*g*. The supernatant was discarded, the pellet was resuspended with 2 ml of phosphate-buffered saline (PBS), and the cell number was counted with a hemocytometer under a microscope.

### RNA purification

Cell suspension (5.0×10^6^ to 1.0×10^7^ cells) was transferred to a 1.5-ml Eppendorf tube and centrifuged for 5 min at 400*g*, 4°C. The supernatant was discarded, and the pellet was resuspended with 1.0 ml of Trizol (Ambion, CAT# 15596026 Thermo Fisher Scientific, Waltham, MA, USA), then sat at room temperature for 5 min to lyse the cells completely. The cell lysate was added with 200 μl of chloroform and shaken well, then sat at room temperature for 5 min. The mixture was centrifuged for 15 min at 12,000*g*, 4°C. The supernatant was carefully transferred into a new Eppendorf tube and then mixed with 500 μl of isopropyl alcohol. After sitting at room temperature for 10 min, the mixture was centrifuged for 10 min at 12,000*g*, 4°C. The supernatant was discarded, and the RNA pellet was washed with 1.0 ml of 75% ethanol (made with absolute ethanol and diethyl pyrocarbonate (DEPC)-treated water). The ethanol was discarded after 5 min of centrifugation at 12,000*g*, 4°C, and the pellet was air-dried at room temperature for 5 min. The RNA was dissolved in 30 μl of DEPC-treated water and kept at -80°C.

### (RT-PCR) Reverse transcription-polymerase chain reaction amplification of TCRβ gene

The first round of PCR amplification of the TCRβ gene was performed with the QIAGEN one-step RT-PCR kit (QIAGEN, CAT# 210212 QIAGEN, Venlo, The Netherlands), using RNA extracted from the peripheral blood mononuclear cells (PBMCs) as a template. PT-PCR contains 1 μg of RNA template, TRTmix primers and Vβmix primers at 0.5 μM each, dNTPs at 0.4 μM each, 1 μl of OneStep RT-PCR enzyme mix, 1× OneStep RT-PCR buffer, and 5 units of RNase inhibitor in a 25-μl volume. The TRTmix primers were a 1:1 mixture of the four primers designed based on functional human T-cell receptor beta constant (TRBC) alleles (**Supplementary Table 3**) while Vβmix primers were a 1:1 mixture of the 36 primers designed based on functional human T-cell receptor beta variable (TRBV) alleles (**Supplementary Table 4**). RT-PCR conditions were as follows: reverse transcription, 50°C, 30 min; PCR activation, 95°C, 15 min; and then, 20 cycles of reactions, 95°C, 30 s; 58°C, 30 s; and 72°C, 30 s.

### PCR amplification of TCRβ gene

The second round of PCR amplification of the TCRβ gene was performed using the first-round RT-PCR product as a template. PCR contains 7.5 μl of the first round RT-PCR product, hTCRCbBCX primer, and Vβmix primers at 0.5 μM each, dNTPs at 0.4 μM each, 1× Gold Taq PCR buffer, 3 μl Golden Taq DNA Polymerase (Toneker Biotech, Shanghai, China, CAT# TK10002), and MgCl_2_ at 2 mM in a 50-μl volume. The Vβmix primers were the same mixture used in the RT-PCR reaction while the hTCRCbBCX primer contains barcodes (**Supplementary Table 5**, the capitalized base pairs indicate the barcodes) before sample identification. The PCR conditions were as follows: activation, 95°C, 10 min and 20 cycles of reactions, 95°C, 30 s; 58°C, 30 s; and 72°C, 30 s. The PCR products were purified with the DNA fragment purification kit (BioMagbeads, Wuxi, China, CAT# BMSX).

### Sequencing of TCRβ gene

The sequencing library was prepared using a Thermal-Fisher Ion Plus Fragment Library Kit (CAT# 4471252 Thermo Fisher Scientific, Waltham, MA, USA), Ion Xpress Barcode Adaptors 1-16Kit (CAT# 4471250 Thermo Fisher Scientific, Waltham, MA, USA), and Agencourt AMPure XP (CAT# A63881 Thermo Fisher Scientific, Waltham, MA, USA), according to the manufacturer’s instruction. Automated template preparation and chip loading were performed on a Thermal-Fisher Ion Chef system using Ion 520/530 ExT-Chef-4rxns &4 Init NEW-For 600bp (CAT# A30670 Thermo Fisher Scientific, Waltham, MA, USA). Then, the TCRβ variable region sequencing was performed on a Thermal-Fisher Ion S5 system with Ion 530 Chip Kit (CAT# A27764).

### Sequencing data analyses

Sequencing results were exported into separated FASTA files for each sample according to the barcodes. The Fasta files were analyzed through a local IgBLAST program using default parameters to assign the used germline Vβ, Jβ gene, complementarity-determining region 3 (CDR3), and CDR3 amino acid sequence for each TCRβ sequence. A TCRβ sequence whose V–D–J junction produced a productive translation of TCRβ peptide with a non-empty CDR3 was defined as “functional.” For comparison studies, 30,000 functional TCRβ sequences were randomly selected from each sample.

### TCRβ diversity index

The diversity index of the TCRβ repertoire was measured by the diversity 50 (D_50_) value, which was defined as the percent of dominant TCRβ CDR3 clonetypes that account for the cumulative 50% of the total TCRβ sequences in a sample. The mathematical formulation of D_50_ calculation is defined as follows:

1. The number of total TCRβ sequences in a sample was defined as N, and the number of all unique TCRβ CDR3 amino acid sequence types was defined as C. The functional TCRβ sequences relevant to each unique CDR3 sequence were defined as N_1_, N_2_, …, N_C_, which were sorted by size as N_1_≥N_2_≥…≥N_C-1_≥N_C_.
2. When (N_1_+N_2_+…+N_H-1_) ≤ 0.5×N while (N_1_+N_2_+…+N_H_) ≥ 0.5×N, D_50_=H/C.

### Statistical analyses

Statistical analysis was performed using the ORIGIN Pro v2021 software (OriginLab Corporation). Non-parametric Mann-Whitney U test was used for comparing two sets of independent variables. Paired sample Wilcoxon signed ranks test was used for comparing two sets of dependent variables.

## Results

### Distinct features of the expressed TCRβ repertoires in the peripheral blood of healthy donors and cancer patients

**Figure 1:**
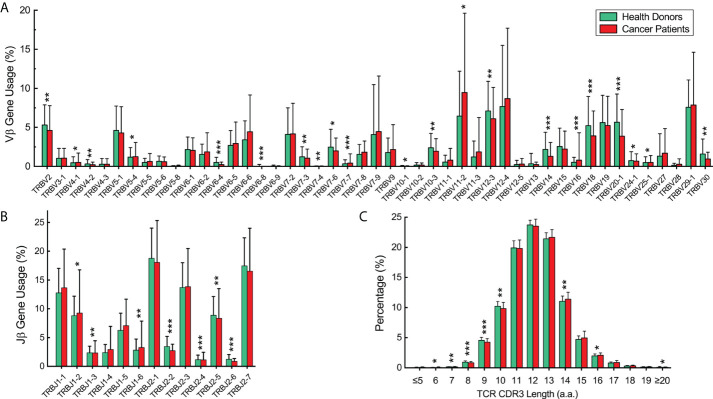
Vβ and Jβ gene usage and complementarity-determining region 3 (CDR3) length distribution of the expressed T-cell receptor (TCR)β repertoires in the peripheral blood of healthy donors and cancer patients. **(A)** Vβ gene usage comparison. The x-axis depicts all functional human TRBV alleles while the y‐axis is the percentage of TCRβ sequences in all repertoires of healthy donors or cancer patients using each TRBV allele. **(B)** Jβ gene usage comparison. The x-axis depicts functional human T-cell Receptor Beta Joining (TRBJ) alleles. **(C)** TCRβ CDR3 amino acid sequence length distribution comparison. The y‐axis is the percentage of TCRβ CDR3 clone types with the corresponding CDR3 length. The error bars indicate standard deviations while the asterisks indicate *p*-values of the Mann-Whitney U test (*p < 0.05; **p < 0.01; ***p < 0.001).

We analyzed the expressed TCRβ repertoires in the peripheral blood samples of 582 healthy donors and 60 cancer patients (**Supplementary Tables 1, 2**). All cancer patients including 41 patients with solid tumor and 19 patients with hematological tumor have received appropriate treatments upon sample collection. Among the 47 functional human Vβ genes, the usages of TRBV2, TRBV4-1, TRBV4-2, TRBV5-4, TRBV6-4, TRBV6-8, TRBV7-3, TRBV7-4, TRBV7-6, TRBV7-7, TRBV10-1, TRBV10-3, TRBV12-3, TRBV14, TRBV18, TRBV20-1, TRBV24-1, TRBV25-1, and TRBV30 in healthy donors are significantly higher than in cancer patients; the usages of TRBV11-2 and TRBV16 in cancer patients are significantly higher than in healthy donors (**Figure 1A**, *p* < 0.05, Mann-Whitney U test). For the functional Jβ genes, the usages of TRBJ1-2, TRBJ1-3, and TRBJ1-6 in healthy donors are significantly higher than in cancer patients; the usages of TRBJ2-2, TRBJ2-4, TRBJ2-5, and TRBJ2-6 in cancer patients are significantly higher than in healthy donors (**Figure 1B**, *p* < 0.05, Mann-Whitney U test). The length distributions of TCRβ CDR3 in healthy donors and cancer patients are similar, peaking at 12-amino acids (**Figure 1C**).

**Figure 2:**
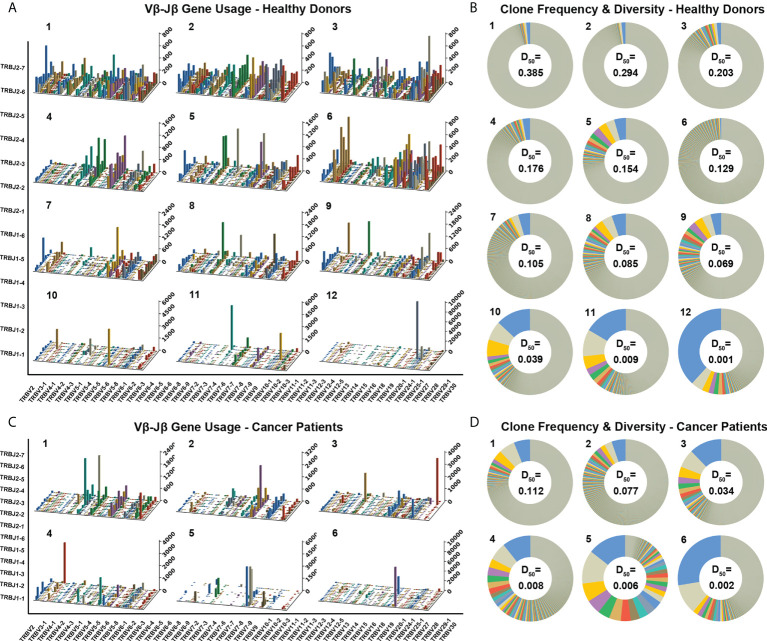
Distinct Vβ–Jβ gene usage combinations, diversities, and clonalities of expressed TCRβ repertoires of healthy donors and cancer patients. **(A)** V–J gene usage combinations for 12 representative repertoires of healthy donors. The x-axis and y-axis depict functional human TRBV and TRBJ alleles, respectively. The z‐axis is the count of TCRβ sequences with a given V–J combination (note the scale of z-axis varies according to changes in the maximum count). **(B)** Clone frequencies and diversities for the 12 representative repertoires (same as the ones shown in panel A, in the same order). The radian of a wedge on the doughnut circle indicates the percentage in the repertoire occupied by functional TCRβ sequences relevant to a given CDR3 clone type (tiny clones are uniformly shown in gray). The D_50_ values for each repertoire are also shown. **(C)** V–J gene usage combinations for six representative repertoires of cancer patients. **(D)** Clone frequencies and diversities for the six repertoires (same as the ones shown in panel C, in the same order).

Each healthy donor or cancer patient has distinct features in the TCR diversity and clonality. Over 60% of healthy donors have relatively high TCR diversities and low clonalities, as visualized by the evenly distributed TCR Vβ–Jβ gene usage combinations and the prevalent low-frequency TCRβ clones through analyzing the TCRβ CDR3 amino acid sequences (**Figure 2A**1–6 showed Vβ–Jβ combinations of six representative repertoires, and **Figure 2B**1–6 showed their TCRβ clone frequencies, respectively); about 30% of healthy donors have relatively low TCR diversities and high clonalities as visualized by the biased TCR Vβ–Jβ gene usage combinations and the appearances of high-frequency TCRβ clones (**Figure 2A**7–12 and **2B**7–12 showed six representative repertoires). Over 80% of cancer patients have relatively low TCR diversities and high clonalities (**Figures 2C, D** showed six representative repertoires). These results indicate that each individual has distinct features of TCRβ repertoire.

### Elder people and cancer patients have low TCR diversities and significant clone expansions

**Figure 3:**
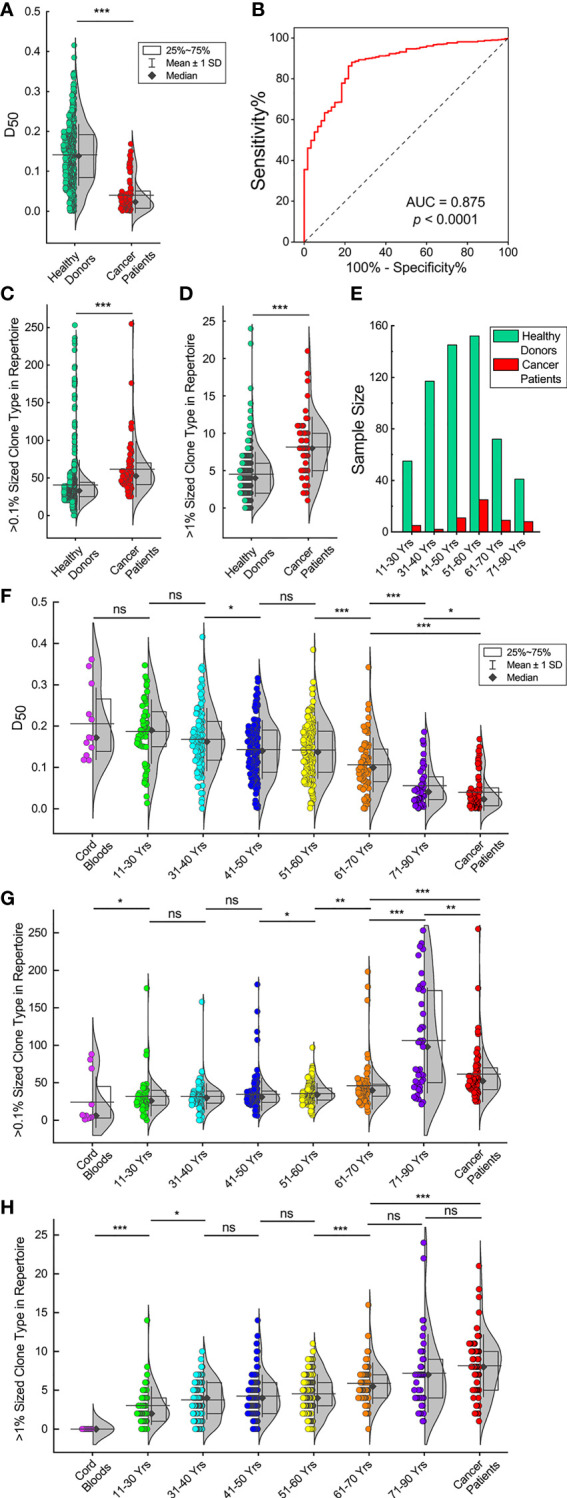
TCRβ repertoires of elder individuals and cancer patients have low diversities and significant clone expansions. **(A)** D_50_ values of 582 healthy donors and 60 cancer patients. The violin plot curve indicates distribution of the data points while the box indicates the majority data chunk (25%~75%); the vertical whiskers depict the standard deviations; the horizontal line depicts the mean value while the black diamond represents the median value (same for all following violin plots). **(B)** Receiver operating characteristic (ROC) curve analysis to distinguish healthy donors from cancer patients according to the D_50_ values. **(C, D)**. TCRβ CDR3 clone types that account for more than 0.1% or 1% of the repertoires for healthy donors and cancer patients. **(E)** Age distributions in both healthy donors and cancer patients. **(F)** D_50_ values of healthy donors in different age groups. D_50_ values of 12 cord blood samples and 60 cancer patients are also shown. **(G, H)**. Over 0.1% or 1% sized TCRβ CDR3 clone types for healthy donors in different age groups, cord blood samples and cancer patients. The asterisks indicate *p*-values of the Mann-Whitney U test (*p < 0.05; **p < 0.01; ***p < 0.001). ns, not significant.

We applied the diversity index (D_50_) as a quantitative measurement of the TCR diversity and clonality. The D_50_ values of the TCRβ repertoires in the 582 healthy donors are significantly higher than in the 60 cancer patients (**Figure 3A**, *p* < 0.001, Mann-Whitney U test). The D_50_ values of these healthy donors show symmetric distribution with a mean value of 0.141 and a close median value of 0.139. In sharp contrast to the healthy donors, the D_50_ values of cancer patients show asymmetric distribution, with a mean value of 0.040 and a deviating median value of 0.023, which means that majority of the samples have D_50_ values far below the average.

The receiver operating characteristic (ROC) curve analysis to distinguish healthy donors from cancer patients according to the D_50_ values has an area under the curve (AUC) of 0.875 (**Figure 3B**, *p* < 0.0001). The ROC analysis has a sensitivity of 67.4% and a specificity of 85.0% if the D_50_ cut-off value is set at 0.10. Thus, we defined that a TCRβ repertoire with a high diversity should have a D_50_ value >0.10; otherwise, it had a low diversity. Following this standard, 67.5% of healthy donors (393 out of 582) had high TCRβ diversities, while 85% of cancer patients (51 out of 60) had low TCRβ diversities. These results are consistent with the Vβ–Jβ usage plots analyses.

One of the factors affecting the TCRβ diversity is the appearance of expanding TCRβ clones. We analyzed TCRβ clones in each individual and considered unique TCRβ CDR3 clone types account for >0.1% of total sequences in an individual repertoire as big TCRβ clones and >1% of total sequences as large TCRβ clones. The results show that cancer patients have significantly higher numbers of big and large TCRβ clones than healthy donors (**Figures 3C, D**, *p* < 0.001, Mann-Whitney U test).

Another factor that may affect the TCRβ diversity is age. Age distributions in both healthy donors and cancer patients peak at 51–60 years old (**Figure 3E**). Since healthy donors had a slightly higher proportion of samples in the younger age groups, the average age is 48.8 for them compared to 54.1 years old for cancer patients (**Supplementary Tables 1, 2**). IA comparison of the D_50_ values of the 12 cord blood samples, healthy donors in different age groups and cancer patients shows that the D_50_ values decrease with increasing age. (**Figure 3F**). The D_50_ values of the cord blood samples are similar to the D_50_ values of individuals at 11–30 years old. The D_50_ values of those ages 11–30 are not significantly different from the D_50_ values of individuals at 31–40 years old. The D_50_ values of those ages 31–40 are significantly higher than the D_50_ values of individuals at 41–50 years old, followed by individuals at 51–60, 61–70, and 71–90 years old (*p* < 0.05, Mann-Whitney U test). It should be pointed out that cancer patients, with an average age of 54.1, have D_50_ values not only far below those of healthy donors at ages 51–60 but even lower than individuals ages 71–90 (*p* < 0.05, Mann-Whitney U test). Such results show clearly that in healthy donors, the TCRβ diversities decline dramatically with increasing age, and cancer patients have very low TCRβ diversities compared to healthy donors.

We also compared the numbers of expanding TCRβ clones among different age groups. The numbers of big clones (>0.1% of total sequences) in individual repertoires elevate with increasing age (**Figure 3G**). The numbers of big clones in cord blood samples are significantly lower than in individuals at 11–30 years old (*p* < 0.05, Mann-Whitney U test). The numbers of big clones in the groups at ages 11–30, 31–40, and 41–50 are similar. The numbers of big clones in those ages 51–60 are significantly higher than in individuals at 41–50 years old (*p* < 0.05, Mann-Whitney U test). The numbers of big clones in those ages 61–70 are significantly higher than in individuals at 51–60 years old (*p* < 0.05, Mann-Whitney U test). The numbers of big clones in those ages 71–90 are significantly higher than in individuals at 61–70 years old (*p* < 0.001, Mann-Whitney U test). The numbers of big clones in cancer patients are significantly higher than in individuals at 61–70 years old (*p* < 0.001, Mann-Whitney U test), while significantly lower than in those aged 71–90 (*p* < 0.01, Mann-Whitney U test).

The numbers of large clones (>1% of total sequences) in individuals at 11–30 years old are significantly higher than in cord blood samples (**Figure 3H**, *p* < 0.001, Mann-Whitney U test). The numbers of large clones in those ages 31–40 are significantly higher than in individuals at 11–30 years old (*p* < 0.05, Mann-Whitney U test). The numbers of large clones in the groups at ages 31–40, 41–50, and 51–60 are similar; the numbers of large clones in those ages 61–70 are significantly higher than in individuals at 51–60 years old (*p* < 0.001, Mann-Whitney U test). The numbers of large clones in the groups at ages 51–60 and 71–90 are similar; the numbers of large clones in cancer patients are significantly higher than in individuals at 61–70 years old (*p* < 0.001, Mann-Whitney U test), but they are not significantly different from those ages 71–90.

These results indicate that the reduction of TCRβ repertoire diversities in elder healthy donors or cancer patients is correlated to increasing numbers of expanding TCRβ clones.

### Loss of shared common TCRβ clones and clone expansions in elder individuals and cancer patients

**Figure 4:**
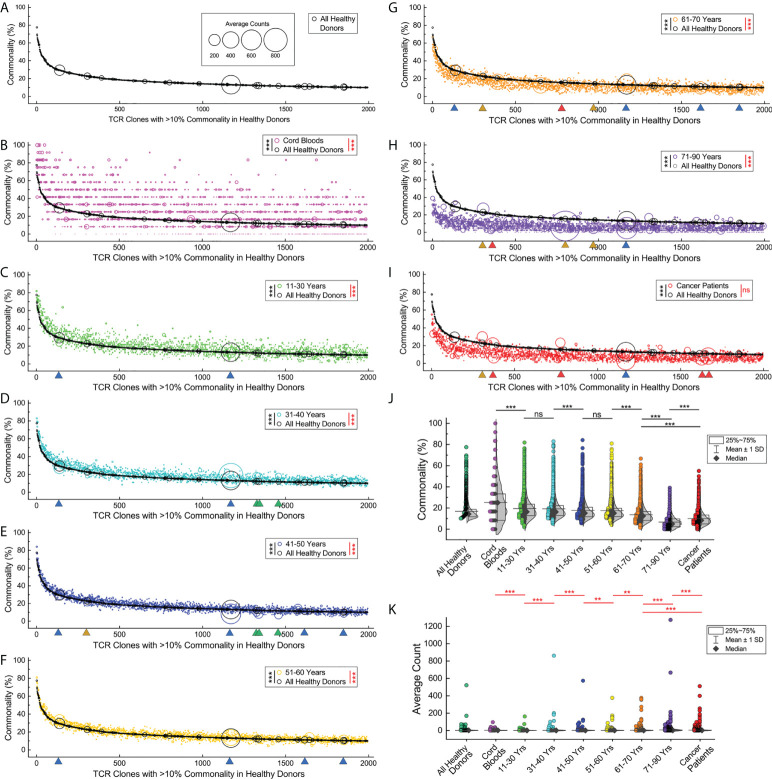
Loss of shared common TCRβ clones and clone expansions in elder individuals and cancer patients. **(A)**. Commonalities and expansions of the high-commonality TCRβ clones (HCTCs) in overall healthy donors. The HCTCs are sorted by their commonalities (x-axis, from high to low). The y‐axis indicates the commonality of each clone type. The area of each symbol represents average clone size of a given clone type (count summary of TCRβ sequences relevant to a given CDR3 clone type in a given subgroup, divided by the times the clone type appears in the subgroup). **(B)** Commonalities and expansions of HCTCs in cord blood samples (magenta symbols) and overall healthy donors (black symbols). **(C–H)**. Commonalities and expansions of HCTCs in healthy donors of different age groups. Green triangles indicate representative clones significantly expanding in younger age groups, while orange or red triangles indicate clones expanding in the elder age groups or cancer patients, and blue triangles indicate clones universally expanding. **(I)** Commonalities and expansions of HCTCs in cancer patients (red symbols). **(J)** Comparison of commonalities of the HCTCs in healthy donors of different age groups, cancer patients, and cord blood samples. **(K)** Comparison of average clone sizes of the HCTCs in healthy donors of different age groups, cancer patients, and cord blood samples. The asterisks beside the panel legends indicate the comparison of commonalities (black asterisks) and average clone sizes (red asterisks) by Wilcoxon signed ranks test (** *p* < 0.01; *** *p* < 0.001). ns, not significant.

It had been reported that different individuals had shared TCRβ clones with identical CDR3 sequences, known as the public TCRβ clones (26). We analyzed the TCRβ clones in the repertoires of the cord blood samples, healthy donors in different age groups, and cancer patients by their commonalities (times a given CDR3 clone type that appears in a subgroup, divided by the sample size of the subgroup) and average counts (count summary of TCRβ sequences relevant to a given CDR3 clone type in a subgroup, divided by the times the clone type appears in the subgroup). We identified 1,994 TCRβ clones that presented in the repertoires of more than 10% of all healthy donors, and we named them the high-commonality TCRβ clones (HCTCs). The commonalities and average counts of these HCTCs in all healthy donors are indicated in the bubble plot, in which the HCTCs are sorted by their commonalities (**Figure 4A**).

Interestingly, a large portion of these HCTCs can be found in cord blood samples with significantly higher commonalities than in overall healthy donors (*p* < 0.001, Wilcoxon signed ranks test), while a small fraction of them do not present in any of the cord blood samples (**Figure 4B**). Most of the HCTCs have relatively low frequencies in cord blood samples, with no profoundly over-sized clone. However, the average sizes of the HCTCs in cord blood samples are statistically larger than in overall healthy donors (*p* < 0.001, Wilcoxon signed ranks test); the HCTCs in the groups at ages 11–30, 31–40, 41–50, and 51–60 have significantly higher commonalities and smaller average counts than in overall healthy donors (**Figures 4C–F**, *p* < 0.001, Wilcoxon signed ranks test). The HCTCs in the groups at ages 61–70 and 71–90 have significantly lower commonalities and higher average counts than in overall healthy donors (**Figures 4G, H**, *p* < 0.001, Wilcoxon signed ranks test). The HCTCs in cancer patients have significantly lower commonalities than in overall healthy donors (**Figure 4I**, *p* < 0.001, Wilcoxon signed ranks test). There are many expanding clones in the cancer patients visualized by the big red circles, although the average sizes of the HCTCs in cancer patients are not statistically significantly higher than in overall healthy donors (*p* > 0.05, Wilcoxon signed ranks test), presumably due to the limited number of samples.

Then, we compared the commonalities of these HCTCs between subgroups (**Figure 4J**). The commonalities of the HCTCs in individuals at 11–30 years old are significantly lower than in cord blood samples (*p* < 0.001, Wilcoxon signed ranks test); the commonalities of the HCTCs in those ages 41–50 are significantly lower than in individuals at 31–40 years old (*p* < 0.001, Wilcoxon signed ranks test). The commonalities of the HCTCs in those ages 61–70 are significantly lower than in individuals at 51–60 years old (*p* < 0.001, Wilcoxon signed ranks test). The commonalities of the HCTCs in individuals at 71–90 years old are significantly lower than in individuals at 61–70 years old (*p* < 0.001, Wilcoxon signed ranks test). It should be pointed out that the commonalities of the HCTCs in cancer patients, with an average age of 54.1, are even lower than in individuals at 61–70 years old, but higher than in those over 71 years of age (*p < 0.001*, Wilcoxon signed ranks test).

On the other hand, some of the HCTCs clonally expand and become big clones with significantly higher frequencies in elder age groups (**Figure 4K**). The average clone sizes of the HCTCs in those ages 31–40 are significantly bigger than in individuals at 11–30 years old (*p* < 0.001, Wilcoxon signed ranks test). The average clone sizes of the HCTCs in those ages 41–50 are significantly bigger than in individuals at 31–40 years old (*p* < 0.001, Wilcoxon signed ranks test). The average clone sizes of the HCTCs in those ages 61–70 are significantly bigger than in individuals at 51–60 years old (*p* < 0.01, Wilcoxon signed ranks test); the average clone sizes of the HCTCs in those ages 71–90 are significantly bigger than in individuals at 61–70 years old (*p* < 0.001, Wilcoxon signed ranks test). Interestingly, the average clone sizes of the HCTCs in cord blood samples are significantly bigger than in individuals at 11–30 years old (*p* < 0.001, Wilcoxon signed ranks test), and the average clone sizes of the HCTCs in those ages 41–50 are significantly bigger than in individuals at 51–60 years old (*p* < 0.01, Wilcoxon signed ranks test). The average clone sizes of the HCTCs in cancer patients are significantly bigger than in individuals at 61–70 years old, while smaller than in those ages 71–90 (*p* < 0.001, Wilcoxon signed ranks test).

These results show that many of these HCTCs appear early in the cord blood, and then they gradually lose their commonalities with increasing age. When comparing prominently expanding clones in different ages, we noticed that some of them expanded universally in both young and old age groups (indicated by blue triangles in **Figure 4C–I**); some clones showed high average counts only in several younger age groups (green triangles), while some significantly expanded in several elder age groups (orange triangles) or in cancer patients (red triangles). These results suggest that the loss of TCRβ clone commonalities in elder individuals and cancer patients is related to clone expansions.

### Expanding and losing commonality, transitions of TCRβ clone characters upon aging and oncogenesis

**Figure 5:**
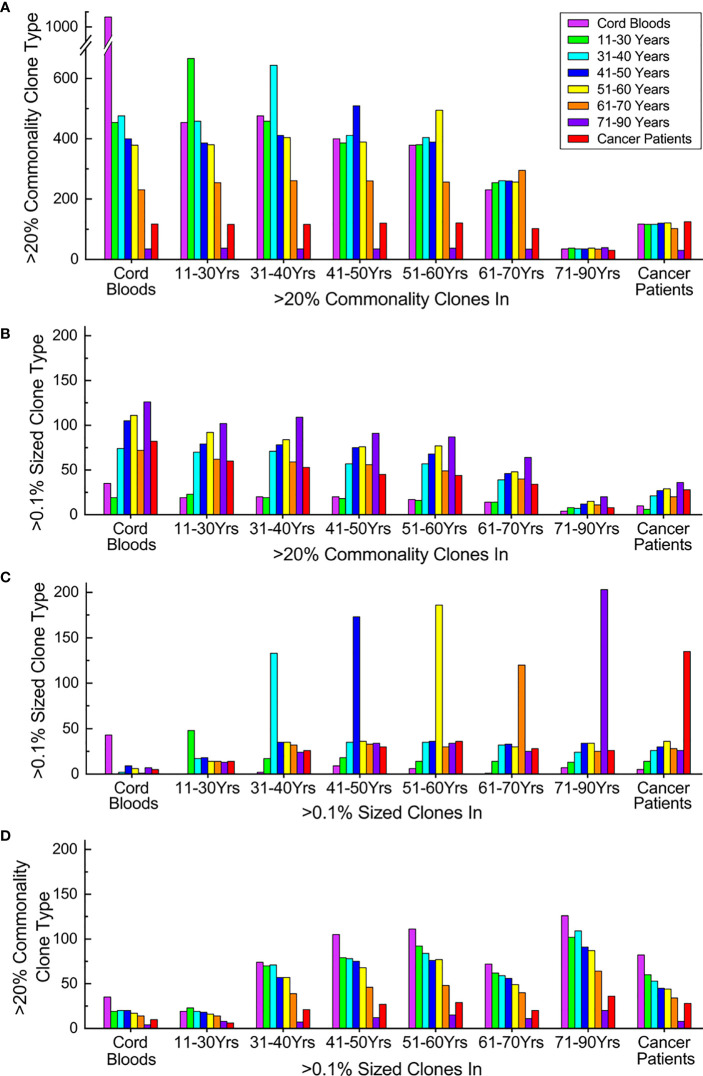
Expanding and losing commonality and transitions of TCRβ clone characters upon aging and oncogenesis. **(A)** Commonality transitions of subgroup high-commonality TCRβ clones (sgHCTCs). Each stack of columns represents sgHCTCs identified in a given subgroup, while each column in the stack indicates the number these sgHCTCs still as >20% commonality clones in different subgroups. **(B)** Expansion transitions of sgHCTCs. Each stack of columns represents sgHCTCs identified in a given subgroup, while each column in the stack indicates the number these sgHCTCs also as >0.1% sized expanding big clones in different subgroups. **(C)** Expansion transitions of expanding big clones. Each stack of columns represents expanding big clones identified in a given subgroup, while each column in the stack indicates the number these big clones still as >0.1% sized clones in different subgroups. **(D)** Commonality transitions of expanding big clones. Each stack of columns represents expanding big clones identified in a given subgroup, while each column in the stack indicates the number of these big clones also as >20% commonality clones in different subgroups. The column colors represent types of given characteristic clones in different subgroups: magenta: cord blood samples, green: 11–30 years old, cyan: 31–40 years old, blue: 41–50 years old, yellow: 51–60 years old, orange: 61–70 years old, violet: 71–90 years old, and red: cancer patients. All panels share the same color mapping. ns, not significant.

To study the transitions of HCTCs between different subgroups, we further defined TCRβ CDR3 sequences shared in >20% of samples in a subgroup as the subgroup high commonality TCRβ clones (sgHCTCs). Then, sgHCTCs in every subgroup are identified, and their commonalities in different subgroups are evaluated (**Figure 5A**). There are a large number of sgHCTCs in cord blood samples and younger individuals, while elder individuals and cancer patients have fewer sgHCTCs. The majority of sgHCTCs identified in a given subgroup are already sgHCTCs in age groups younger than the one in which they are identified. However, they gradually lose their commonalities in elder age groups or in cancer patients. Considering the huge theoretic TCRβ clone types, the shared common TCRβ clones among a given group of individuals are an unlikely coincidence of random V(D)J recombination during T lymphocyte development. Such commonalities might be built up during immune responses and immune memories against certain common antigens. These results suggest that people built their adaptive immunity memory pool with many common responses when they were young and then gradually lost these shared immune memories when they got older or developed tumors.

Some of these sgHCTCs are found to be expanding big clones (accounting for >0.1% of total sequences in the repertoire of at least one individual in a given subgroup) as well. The numbers of sgHCTCs identified in every subgroup also characterized as big clones in a different subgroup are evaluated (**Figure 5B**). The number of expanding sgHCTCs increases upon aging. These results further suggest that the loss of TCRβ clone commonalities is related to clone expansions.

Expanding big clones in every subgroup are identified, and then their expansions in different subgroups are evaluated (**Figure 5C**). Cord blood samples and younger individuals have fewer expanding clone types, while elder individuals and cancer patients have much more. Unlike the sgHCTCs, the majority of the expanding clones identified in a given subgroup are not big clones in other subgroups.

Some of these big clones are found to be sgHCTCs as well. The numbers of big clones identified in every subgroup also characterized as sgHCTCs in each subgroup are evaluated (**Figure 5D**). Regardless of which subgroup the big clones are identified, a large portion (over 40%) of them are characterized as sgHCTCs in cord blood samples or in the youngest individuals, while such commonalities are gradually lost in elder individuals or in cancer patients.

These results suggest that many clone expansions occurring in young individuals might represent immune responses against various common antigens. When these antigens are eliminated, the expanded clones regress while the acquired common immune memories are maintained in the repertoire as high-commonality clones. However, many of these once public clones recording common immune responses are absent in the repertoires when people got old.

In summary, TCRβ clone expansions in elder people and cancer patients are accompanied by loss of TCRβ clone commonalities, which may indicate impaired adaptive immunity.

### Correlation of TCRβ clone commonality with repertoire diversity

**Figure 6:**
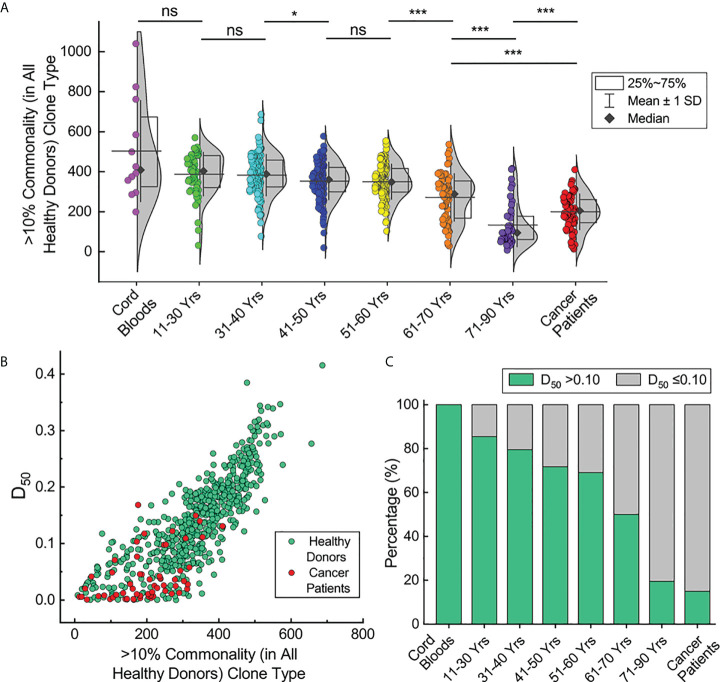
Correlation of TCRβ clone commonality with repertoire diversity. **(A)** HCTC types in individual repertoires of the cord blood samples, healthy donors in different age groups and cancer patients. **(B)** Correlation of HCTC types with repertoire diversities for repertoires of healthy donors and cancer patients. **(C)** Percentage of repertoires with >0.10 diversity index values in each subgroup. The asterisks indicate *p*-values of the Mann-Whitney U test (*p < 0.05; ***p < 0.001). ns, not significant.

Next, the types of HCTCs in the repertoires of the cord blood samples, healthy donors in different age groups, and cancer patients were evaluated (**Figure 6A**). The types of HCTCs in those ages 41–50 are significantly lower than in individuals at 31–40 years old (*p* < 0.05, Mann-Whitney U test). The types of HCTCs in those ages 61–70 are significantly lower than in individuals at 51–60 years old (*p* < 0.001, Mann-Whitney U test). The types of HCTCs in those ages 71–90 are significantly lower than in individuals at 61–70 years old (*p* < 0.001, Mann-Whitney U test). The types of HCTCs in cancer patients are significantly lower than in individuals aged 61–70 years old, but higher than in those aged 71–90 (*p* < 0.001, Mann-Whitney U test). The types of HCTCs in individual repertoires are clearly correlated with the TCR diversity index (D_50_) values (**Figure 6B**). These results suggest that senility and cancer lead to loss of TCRβ repertoire diversity and commonality, probably due to clone expansions caused by immune responses related to aging or oncogenesis.

According to the cut-off value of 0.10 for the D_50_ values we defined previously, the fractions of individuals with high D_50_ values decrease upon aging (**Figure 6C**). There are over 80% of the young healthy donors with D_50_ values greater than 0.10, while only about 20% of healthy donors ages 71–90 have D_50_ values over 0.10. All cord blood samples have D_50_ values over 0.10, while only 15% of cancer patients have D_50_ values over 0.10. Given the common recognition that elder individuals and cancer patients have deteriorated immunities, these results suggest that the TCR diversity index (D_50_) may reflect the potency of the adaptive immune system in different individuals.

## Discussion

Immune repertoire studies provide important information regarding the development and function of the immune system (13, 15). Previous analyses of TCR repertoires frequently observed a variety of differences between two groups of samples, such as differences in Vβ and Jβ gene usage frequencies, CDR3 length distributions, or differential amino acid usage in their CDR3. TCR repertoire diversities are correlated to the pathogenesis or prognosis of various diseases (23, 24). Cancer patients had increasingly biased TCR repertoire with age in tumor-infiltrating lymphocytes, but not in the peripheral blood lymphocytes (25). Studies with a limited number of samples also showed that the TCR repertoire diversity linearly decreased with age (19, 20). However, without thorough analyses of a large number of samples, it is not clear how to interpret the differences between the repertoires of two small groups of samples and how to provide a useful guide for clinical practice.

In this study, we compared the expressed TCRβ repertoires from 582 healthy donors, 12 cord blood samples, and 60 cancer patients. In total, 19,619,328 functional TCRβ gene sequences and 6,332,812 unique TCRβ CDR3 sequences were processed and analyzed. This large-sized TCR database enabled us to reveal fundamental changes in the adaptive immune system. Indeed, we found that each individual has a distinct TCR repertoire, with differential usage of Vβ, Dβ, and Jβ genes, different TCR diversity index values, and different dominant clones. Importantly, the TCR diversity index values in elder individuals are significantly lower than those in younger individuals, and the TCR diversity index values in cancer patients are significantly lower than those in the healthy donors. Such differences are correlated with the increasing frequencies of large oligo clones in elder individuals and cancer patients.

The generation of diversified BCR or TCR repertoires is the foundation of adaptive immunity. However, there are many factors that will affect the diversity of the TCR repertoire. First, thymic involution in humans starts at age 29, and there are almost no detectable newly generated T cells from the thymus after 50 years of age (22). The thymus is the only organ producing T cells. The thymus starts to wither after puberty, which means we will progressively lose the ability to generate naive T lymphocytes (27, 28). Our results clearly show that the TCR diversity index values in healthy donors decline with increasing age. Second, during immune responses against various invading microbes or malignant cells, T cells with antigen-specific TCRs will be activated and proliferate, resulting in the expansion of TCR clones. Clone expansion will dramatically change the TCR diversity index. Normally, after the elimination of antigens, the expanded TCR clones will decline, and only a small portion of these expanded TCR clones will be kept in the memory T cells. Thus, the TCR repertoire in the human body is like a recorder of the immune system. Since each individual may have experienced different diseases in life, the recorded dominant clones in each individual might be different. Third, activation of T cells can eventually lead to cell death. In the case of chronic inflammation, certain TCR clones may be completely eliminated. Such effect is more dramatic in elder individuals, since there are no newly produced T cells from the thymus. In cancer patients, with long-term stimulation from tumor antigens, chemotherapy, and radiotherapy, tumor-specific T cells may be eliminated or exhausted, resulting in very low TCR diversity index values. Moreover, the circulating T cells are composed of various T cell subsets. The differences of TCR diversities and clonalities observed in different samples can either originate from different TCR repertoires in each subset or from different compositions of different cell subsets. Detailed analyses and comparisons of the expressed TCR genes, the diversities of the TCR repertoire, and shared clones among different T-cell subsets in each individual, especially in individuals with different diseases, are worth studying in the future. On the other hand, analyzing TCR repertoires of different T-cell subsets requires FACS sorting of fresh blood samples, which can be completed in a research laboratory for a limited number of samples, but this is not suitable for analyzing a large number of samples in every hospital, especially in rural areas.

It has been reported that a broad TCR repertoire with up to thousands of clonal types is used to recognize a single peptide from the influenza A virus (29, 30). This indicates that we need thousands or millions of TCRs to combat even a single virus or tumor. In this regard, elder people who have lost many TCR clone types, either due to low production of naive T cells or death of memory T cells, have only limited TCRs to be used when facing pathogenic invasion. This may also be the reason for the presence of large oligo TCR clones observed in elder people or cancer patients. Based on these observations, we propose that the TCR diversity index and clonality in the peripheral blood be used to evaluate adaptive immunity.

Thymic involution upon aging is related to rising disease incidence (31). For elder people, the T-cell homeostasis is mainly maintained by peripheral T-cell proliferation, which is associated with partial differentiation and oligoclonal memory inflation as well as loss of memory T cells, with rising disease incidence upon aging (31, 32). Elder individuals have difficulty generating immune memories against new antigens or boosting existing immune memories (33–36). Previous analyses showed that although people progressively lost naive CD8^+^ T cells with age, the absolute numbers of naive CD4^+^ T cells in peripheral blood remained stable (37, 38). In another study, despite the fact that the number of unique TCR clones of both CD4^+^ and CD8^+^ naive T cells in the repertoires of 70-year-old individuals declined by a factor of 2 to 5 compared to the repertoires of 30-year-old individuals, the repertoire diversities were still very high (39). The loss of high-commonality TCRβ clones in the elder individuals and cancer patients observed in this study provides a clear picture of such changes. With increasing numbers of clonally expanded TCR sequences, elder individuals and cancer patients may not only lose their memory T cells acquired in immune responses against known pathogens or antigens, but they may also lose diverged naive T-cell clones that potentially target emerging neo-antigens generated by the malignant aging cells or new invading microbes. The commonalities of TCRβ clones in cancer patients are significantly lower than in healthy donors. The repertoires of elder individuals or cancer patients have significantly fewer high-commonality TCRβ clone types than younger people. In the cancer-patient group, there are more people with larger-sized TCRβ clones than in healthy donors; in the elder-individual group, there are more people with larger-sized TCRβ clones than in the youth. These findings indicate that the loss of TCR diversities is due to clone expansions.

On the other hand, our detailed analyses of the TCRβ clone types showed high commonalities among healthy donors. The majority of these TCRβ clones are also found in cord blood samples with significantly higher commonalities than in healthy donors. Meanwhile, the commonalities for these TCRβ clones gradually decrease with increasing age. A large portion of expanding big clones in the elders or cancer patients may be due to individualized immune responses in each person, either from prolonged inflammations or against various endogenous antigens such as autoimmune responses or malignancies (40). These findings are consistent with the latest single-cell sequencing study that the expanding T-cell clones in aged mice differed in the specific TCRαβ chains, which meant there were no shared aged antigens (41, 42). It has been reported that the long-term stability of T-cell clonal composition with remarkable fractions of T cells overlapped at different ages within each individual, and many of the top-sized TCRβ CDR3 clones persisted for more than 20 years (43). Here, we observed more profound changes in TCRβ clone commonalities when compared with the TCRβ repertoires from a larger number of individuals, covering a broader range of ages. It had been reported that continuous immune responses against viral infection led to the loss of CD8^+^ memory T-cell diversity (44), while other studies showed that thymic involution might initiate accumulation of specific dysregulated CD4^+^ T cells (45). It had also been shown that the fraction of shared naive CD4 T cell clone types grew with aging (46).

In summary, we find that the TCR diversity index (D_50_) reduces with aging and disease, and elder individuals and cancer patients have very low D_50_ values. Consistently, in elder people and cancer patients, the commonalities of TCRβ clones reduce and the number of large TCR clones increase. Based on these results, we propose the hypothesis that the TCR diversity index can be used as a simple method to compare the adaptive immunities of different people or to track the change of immune statuses in the same person. Certainly, we will continue this line of studies in more detail in the future, such as determining if the TCR diversity index values correlate with the risk of cancer or infection in healthy donors, or correlate with the outcomes of different treatments in cancer patients, or if the TCR diversity index values change during immune therapy. We believe that analyzing the diversity and clonality of the peripheral TCR repertoire may offer a simple method for evaluating and comparing the adaptive immune system, and this simple method will be very useful in many immunological studies and clinical practice in the future.

## Data availability statement

The raw data supporting the conclusions of this article will be made available by the authors, without undue reservation.

## Ethics statement

The studies involving human participants were reviewed and approved by Medical Ethics Committee of Sichuan Provincial People’s Hospital. The patients/participants provided their written informed consent to participate in this study.

## Author contributions

ZZ, YL, YZ designed and directed the study; YZ, PS, XW, XZ conducted the experiments; ZZ, XY, YZ performed the sequence analyses; ZZ, YL, YZ, PS, LY, SY, SX organized patient recruitment and sample collection. All of the authors worked together to write the manuscript. YZ and XY contributed equally to this study. All authors contributed to the article and approved the submitted version.

## Funding

This work was supported by Sichuan Provincial Department of Science and Technology Grant 2020YFS0016 to ZZ as a part of the COVID-19 Responding Key Research and Development Program and by Start-up Research Grants provided by Sichuan Provincial People’s Hospital and University of Electronic Science and Technology of China to ZZ and YZ.

## Acknowledgments

We are thankful to our colleagues in the Department of Health Management & the Institute of Health Management who provided expertise that greatly assisted the research.

## Conflict of interest

ZZ is the founder of Chengdu ExAb Biotechnology, LTD. Authors XY, XW, XZ, and SY were employed by Chengdu ExAb Biotechnology, LTD.

The remaining authors declare that the research was conducted in the absence of any commercial or financial relationships that could be construed as a potential conflict of interest.

## Publisher’s note

All claims expressed in this article are solely those of the authors and do not necessarily represent those of their affiliated organizations, or those of the publisher, the editors and the reviewers. Any product that may be evaluated in this article, or claim that may be made by its manufacturer, is not guaranteed or endorsed by the publisher.
