# Supplementary material for: Evaluation and comparison of adaptive immunity through analyzing the diversities and clonalities of T-cell receptor repertoires in the peripheral blood
Source: Front Immunol. 2022 Sep 8;13:916430. doi: 10.3389/fimmu.2022.916430 (PMC9493076; doi:10.3389/fimmu.2022.916430)
Supplement: Supplementary file 1 [file Table_1.docx]

**Supplementary Tables**

**Supplementary Table 1. Information of recruited healthy donors.**

| Healthy Donors | | | | | | | | | | | | | | | | | | | |
| --- | --- | --- | --- | --- | --- | --- | --- | --- | --- | --- | --- | --- | --- | --- | --- | --- | --- | --- | --- |
|  | All | 11-30 Years | | | 31-40 Years | | | 41-50 Years | | | 51-60 Years | | | 61-70 Years | | | 71-90 Years | | |
| Total | 582 | 55 | | | 117 | | | 145 | | | 152 | | | 72 | | | 41 | | |
| Male | 285 | 26 | | | 58 | | | 71 | | | 77 | | | 35 | | | 19 | | |
| Female | 296 | 29 | | | 59 | | | 74 | | | 75 | | | 37 | | | 22 | | |
| Avrg* Age | 48.8 | 26.3 | | | 34.9 | | | 45.7 | | | 55.3 | | | 64.3 | | | 78.2 | | |
| Detail List | | Age | Gndr | Prov | Age | Gndr | Prov | Age | Gndr | Prov | Age | Gndr | Prov | Age | Gndr | Prov | Age | Gndr | Prov |
|  |  | 20 | F | HN | 31 | M | HN | 41 | F | TJ | 51 | M | HN | 61 | M | SC | 71 | M | SC |
|  |  | 21 | M | SC | 31 | F | HN | 41 | M | SC | 51 | M | SC | 61 | M | BJ | 72 | M | SC |
|  |  | 21 | M | SC | 31 | F | HN | 41 | M | YN | 51 | M | TJ | 61 | M | SC | 72 | M | SC |
|  |  | 21 | F | HN | 31 | M | TJ | 41 | M | HN | 51 | F | HN | 61 | F | SC | 72 | M | SC |
|  |  | 21 | M | SC | 31 | M | HN | 41 | F | HN | 51 | F | HN | 61 | F | SC | 72 | M | TJ |
|  |  | 22 | F | SC | 31 | F | TJ | 41 | F | HN | 51 | M | SX | 61 | F | HN | 72 | M | SC |
|  |  | 22 | F | SX | 31 | M | SC | 41 | F | HN | 51 | M | SC | 61 | M | YN | 72 | F | SC |
|  |  | 23 | F | TJ | 31 | F | HN | 41 | F | TJ | 51 | M | SC | 61 | M | HN | 72 | F | SC |
|  |  | 23 | F | HN | 31 | M | SH | 41 | M | HN | 51 | M | SC | 61 | F | HN | 72 | F | SC |
|  |  | 24 | F | HN | 31 | F | HN | 41 | M | HN | 51 | F | ZJ | 61 | M | TJ | 73 | F | SC |
|  |  | 24 | M | TJ | 31 | F | TJ | 42 | M | SC | 51 | M | HN | 61 | F | SC | 73 | M | SC |
|  |  | 24 | M | SC | 31 | M | SC | 42 | F | HN | 51 | F | HN | 62 | F | SC | 74 | F | SC |
|  |  | 24 | F | HN | 31 | F | HN | 42 | M | SC | 51 | M | SC | 62 | M | SC | 74 | F | TJ |
|  |  | 24 | M | SC | 31 | M | SC | 42 | F | ZJ | 51 | M | SC | 62 | F | SC | 74 | F | SC |
|  |  | 25 | M | SC | 31 | M | HN | 42 | F | HN | 51 | M | SC | 62 | F | SC | 74 | M | SC |
|  |  | 25 | M | TJ | 31 | F | HN | 42 | M | SC | 51 | M | HN | 62 | F | SC | 75 | M | SC |
|  |  | 25 | M | SC | 31 | F | TJ | 42 | F | SH | 51 | F | SC | 62 | F | SC | 76 | F | SC |
|  |  | 25 | F | HN | 31 | M | BJ | 42 | F | SC | 52 | M | SC | 62 | F | HN | 76 | M | SC |
|  |  | 25 | F | TJ | 31 | F | TJ | 42 | F | TJ | 52 | F | SX | 62 | M | SC | 76 | F | SC |
|  |  | 25 | F | SC | 31 | M | TJ | 42 | M | ZJ | 52 | M | TJ | 62 | M | SC | 77 | F | SC |
|  |  | 25 | F | TJ | 32 | F | TJ | 42 | F | HN | 52 | M | SC | 62 | M | TJ | 77 | F | SC |
|  |  | 25 | F | SC | 32 | F | GD | 42 | M | SC | 52 | M | SC | 62 | F | HN | 79 | F | SC |
|  |  | 25 | M | SH | 32 | M | HN | 42 | M | SH | 52 | F | HN | 62 | M | SC | 79 | F | SC |
|  |  | 26 | F | HN | 32 | F | TJ | 42 | F | SC | 52 | F | SC | 62 | F | SC | 79 | M | SC |
|  |  | 26 | F | TJ | 32 | M | HN | 42 | F | HN | 52 | F | SH | 62 | M | SC | 79 | F | HN |
|  |  | 26 | F | HN | 32 | F | HN | 42 | M | TJ | 53 | F | HN | 63 | M | SC | 79 | M | SC |
|  |  | 26 | F | SC | 32 | M | SC | 42 | F | SX | 53 | M | TJ | 63 | M | SC | 80 | F | HN |
|  |  | 26 | F | TJ | 32 | F | SC | 42 | M | SC | 53 | F | TJ | 63 | F | HN | 81 | F | SC |
|  |  | 27 | M | SC | 32 | F | TJ | 43 | F | TJ | 53 | F | SC | 63 | M | SC | 81 | M | SC |
|  |  | 27 | M | HN | 32 | M | HN | 43 | M | HN | 53 | F | GD | 63 | M | SC | 83 | F | SC |
|  |  | 27 | M | SC | 32 | M | HN | 43 | F | HN | 53 | F | SC | 63 | M | SC | 83 | M | SC |
|  |  | 27 | F | TJ | 32 | M | HN | 43 | F | HN | 53 | M | SC | 63 | F | TJ | 83 | M | SC |
|  |  | 27 | F | SH | 32 | M | SC | 43 | F | TJ | 53 | F | GD | 63 | M | SC | 84 | F | SC |
|  |  | 28 | F | HN | 32 | M | SC | 43 | M | BJ | 53 | F | TJ | 63 | M | SC | 84 | M | SC |
|  |  | 28 | F | TJ | 32 | M | SH | 43 | F | TJ | 53 | F | HN | 63 | F | SC | 85 | F | SC |
|  |  | 28 | M | SC | 33 | F | HN | 43 | F | SC | 53 | F | HN | 63 | F | HN | 85 | M | SC |
|  |  | 28 | M | SC | 33 | M | HN | 43 | F | ZJ | 53 | F | HN | 64 | F | HN | 86 | F | SC |
|  |  | 28 | F | HN | 33 | F | HN | 43 | M | SC | 53 | M | SC | 64 | F | TJ | 87 | F | SC |
|  |  | 29 | M | HN | 33 | F | HN | 43 | M | TJ | 53 | F | SC | 64 | F | SC | 88 | M | HN |
|  |  | 29 | M | HN | 33 | F | SC | 43 | F | YN | 53 | M | SC | 64 | M | SC | 88 | M | SC |
|  |  | 29 | F | HN | 33 | M | HN | 43 | F | SC | 53 | M | SC | 64 | F | TJ | 89 | F | SC |
|  |  | 29 | M | TJ | 33 | F | SC | 43 | M | SC | 53 | M | SX | 64 | F | SH |  |  |  |
|  |  | 29 | M | TJ | 33 | F | HN | 43 | F | SC | 53 | M | HN | 64 | M | SC |  |  |  |
|  |  | 29 | M | TJ | 33 | F | TJ | 43 | M | TJ | 53 | M | SC | 65 | M | ZJ |  |  |  |
|  |  | 29 | M | TJ | 33 | F | HN | 43 | F | HN | 53 | F | TJ | 65 | M | SC |  |  |  |
|  |  | 30 | F | HN | 33 | F | SX | 43 | M | HN | 53 | F | SX | 65 | F | TJ |  |  |  |
|  |  | 30 | M | TJ | 33 | M | HN | 44 | M | HN | 53 | F | HN | 65 | M | SH |  |  |  |
|  |  | 30 | M | SC | 33 | M | SC | 44 | F | SC | 54 | F | TJ | 65 | M | SC |  |  |  |
|  |  | 30 | F | HN | 33 | M | HN | 44 | F | TJ | 54 | F | YN | 65 | F | TJ |  |  |  |
|  |  | 30 | F | SH | 35 | F | HN | 44 | M | SC | 54 | M | SC | 66 | F | TJ |  |  |  |
|  |  | 30 | F | SC | 34 | F | SC | 44 | M | HN | 54 | M | SC | 66 | M | SH |  |  |  |
|  |  | 30 | M | HN | 34 | M | SH | 44 | M | HN | 54 | M | SC | 66 | F | HN |  |  |  |
|  |  | 30 | F | HN | 34 | M | HN | 44 | M | HN | 54 | M | SC | 66 | M | HN |  |  |  |
|  |  | 30 | M | SH | 34 | M | HN | 44 | M | ZJ | 54 | M | TJ | 66 | F | SC |  |  |  |
|  |  | 30 | M | SC | 34 | F | SC | 44 | M | HN | 54 | M | SC | 67 | F | SC |  |  |  |
|  |  |  |  |  | 35 | F | SH | 44 | M | HN | 54 | M | SC | 67 | F | TJ |  |  |  |
|  |  |  |  |  | 35 | M | HN | 44 | F | SC | 54 | M | HN | 67 | F | HN |  |  |  |
|  |  |  |  |  | 35 | F | HN | 44 | F | HN | 55 | M | HN | 67 | M | SH |  |  |  |
|  |  |  |  |  | 36 | F | HN | 44 | M | BJ | 55 | F | ZJ | 68 | F | SX |  |  |  |
|  |  |  |  |  | 35 | M | HN | 44 | M | SC | 55 | F | ZJ | 68 | M | SH |  |  |  |
|  |  |  |  |  | 35 | F | TJ | 44 | M | SC | 55 | F | ZJ | 68 | M | TJ |  |  |  |
|  |  |  |  |  | 35 | M | SC | 44 | F | HN | 55 | M | SH | 68 | F | TJ |  |  |  |
|  |  |  |  |  | 35 | M | HN | 45 | F | HN | 55 | F | HN | 68 | M | SC |  |  |  |
|  |  |  |  |  | 35 | F | TJ | 45 | F | ZJ | 55 | F | BJ | 69 | M | SC |  |  |  |
|  |  |  |  |  | 35 | M | HN | 45 | M | HN | 55 | M | SH | 69 | F | GD |  |  |  |
|  |  |  |  |  | 35 | M | SC | 45 | M | HN | 55 | F | SC | 69 | F | SC |  |  |  |
|  |  |  |  |  | 35 | M | HN | 45 | F | SC | 54 | F | TJ | 69 | F | SC |  |  |  |
|  |  |  |  |  | 35 | M | SX | 45 | M | SC | 55 | F | HN | 69 | M | TJ |  |  |  |
|  |  |  |  |  | 35 | M | TJ | 45 | M | TJ | 55 | M | HN | 70 | F | TJ |  |  |  |
|  |  |  |  |  | 35 | F | SC | 46 | M | HN | 55 | M | BJ | 70 | M | SC |  |  |  |
|  |  |  |  |  | 35 | F | TJ | 46 | F | TJ | 55 | F | HN | 70 | M | HN |  |  |  |
|  |  |  |  |  | 36 | F | HN | 46 | F | TJ | 55 | F | ZJ | 70 | F | SC |  |  |  |
|  |  |  |  |  | 36 | M | TJ | 46 | F | TJ | 55 | M | SC |  |  |  |  |  |  |
|  |  |  |  |  | 36 | F | TJ | 46 | M | SC | 55 | F | HN |  |  |  |  |  |  |
|  |  |  |  |  | 36 | M | SC | 46 | M | TJ | 55 | M | SC |  |  |  |  |  |  |
|  |  |  |  |  | 36 | F | SH | 46 | F | HN | 55 | M | SC |  |  |  |  |  |  |
|  |  |  |  |  | 36 | F | SX | 46 | M | SX | 55 | F | TJ |  |  |  |  |  |  |
|  |  |  |  |  | 36 | M | SC | 46 | F | SX | 55 | M | HN |  |  |  |  |  |  |
|  |  |  |  |  | 37 | F | HN | 46 | M | SC | 56 | M | TJ |  |  |  |  |  |  |
|  |  |  |  |  | 37 | M | HN | 46 | M | HN | 56 | F | TJ |  |  |  |  |  |  |
|  |  |  |  |  | 37 | F | HN | 46 | F | HN | 56 | M | TJ |  |  |  |  |  |  |
|  |  |  |  |  | 37 | F | TJ | 47 | M | HN | 56 | F | TJ |  |  |  |  |  |  |
|  |  |  |  |  | 37 | F | GD | 47 | M | HN | 56 | M | SC |  |  |  |  |  |  |
|  |  |  |  |  | 37 | F | SC | 47 | F | SC | 56 | F | HN |  |  |  |  |  |  |
|  |  |  |  |  | 37 | F | SX | 47 | F | HN | 56 | F | HN |  |  |  |  |  |  |
|  |  |  |  |  | 37 | F | SX | 47 | M | TJ | 56 | F | HN |  |  |  |  |  |  |
|  |  |  |  |  | 37 | F | HN | 47 | F | HN | 56 | F | SX |  |  |  |  |  |  |
|  |  |  |  |  | 37 | M | SC | 47 | M | SC | 56 | F | HN |  |  |  |  |  |  |
|  |  |  |  |  | 38 | F | HN | 47 | M | SC | 56 | M | TJ |  |  |  |  |  |  |
|  |  |  |  |  | 38 | M | TJ | 47 | M | TJ | 56 | M | HN |  |  |  |  |  |  |
|  |  |  |  |  | 38 | M | HN | 47 | M | SC | 56 | M | HN |  |  |  |  |  |  |
|  |  |  |  |  | 38 | F | HN | 47 | F | TJ | 56 | F | TJ |  |  |  |  |  |  |
|  |  |  |  |  | 38 | M | HN | 48 | F | HN | 56 | F | SC |  |  |  |  |  |  |
|  |  |  |  |  | 38 | F | SC | 48 | M | SC | 56 | M | SC |  |  |  |  |  |  |
|  |  |  |  |  | 38 | M | TJ | 48 | F | SC | 56 | M | SC |  |  |  |  |  |  |
|  |  |  |  |  | 38 | F | SC | 48 | M | SC | 56 | M | HN |  |  |  |  |  |  |
|  |  |  |  |  | 38 | M | SH | 48 | M | TJ | 56 | F | TJ |  |  |  |  |  |  |
|  |  |  |  |  | 38 | M | TJ | 48 | M | SX | 56 | F | TJ |  |  |  |  |  |  |
|  |  |  |  |  | 40 | F | HN | 48 | M | HN | 56 | M | SX |  |  |  |  |  |  |
|  |  |  |  |  | 39 | F | HN | 48 | F | SC | 56 | M | HN |  |  |  |  |  |  |
|  |  |  |  |  | 39 | F | HN | 48 | M | SC | 56 | M | SC |  |  |  |  |  |  |
|  |  |  |  |  | 39 | M | TJ | 48 | F | TJ | 56 | M | TJ |  |  |  |  |  |  |
|  |  |  |  |  | 39 | M | HN | 48 | F | SC | 56 | M | HN |  |  |  |  |  |  |
|  |  |  |  |  | 39 | F | TJ | 48 | F | SC | 56 | M | SC |  |  |  |  |  |  |
|  |  |  |  |  | 39 | M | SC | 48 | F | HN | 57 | F | HN |  |  |  |  |  |  |
|  |  |  |  |  | 39 | M | HN | 48 | F | ZJ | 58 | M | HN |  |  |  |  |  |  |
|  |  |  |  |  | 39 | F | SH | 48 | M | HN | 57 | F | HN |  |  |  |  |  |  |
|  |  |  |  |  | 39 | M | HN | 49 | F | SC | 57 | F | ZJ |  |  |  |  |  |  |
|  |  |  |  |  | 40 | F | TJ | 49 | M | SC | 57 | M | SC |  |  |  |  |  |  |
|  |  |  |  |  | 40 | M | SC | 49 | M | HN | 57 | F | HN |  |  |  |  |  |  |
|  |  |  |  |  | 40 | F | HN | 49 | M | SC | 57 | F | HN |  |  |  |  |  |  |
|  |  |  |  |  | 40 | M | SC | 49 | F | HN | 57 | F | HN |  |  |  |  |  |  |
|  |  |  |  |  | 40 | M | HN | 49 | F | HN | 57 | M | SC |  |  |  |  |  |  |
|  |  |  |  |  | 40 | M | HN | 49 | F | TJ | 57 | M | HN |  |  |  |  |  |  |
|  |  |  |  |  | 40 | F | TJ | 49 | F | HN | 57 | M | BJ |  |  |  |  |  |  |
|  |  |  |  |  | 40 | M | BJ | 49 | F | SC | 57 | M | HN |  |  |  |  |  |  |
|  |  |  |  |  | 40 | M | TJ | 49 | F | HN | 57 | F | TJ |  |  |  |  |  |  |
|  |  |  |  |  |  |  |  | 49 | M | HN | 57 | M | SC |  |  |  |  |  |  |
|  |  |  |  |  |  |  |  | 49 | F | SC | 57 | F | HN |  |  |  |  |  |  |
|  |  |  |  |  |  |  |  | 49 | M | HN | 57 | M | YN |  |  |  |  |  |  |
|  |  |  |  |  |  |  |  | 49 | F | HN | 57 | M | SC |  |  |  |  |  |  |
|  |  |  |  |  |  |  |  | 49 | M | HN | 57 | M | YN |  |  |  |  |  |  |
|  |  |  |  |  |  |  |  | 49 | F | TJ | 57 | M | HN |  |  |  |  |  |  |
|  |  |  |  |  |  |  |  | 50 | F | TJ | 58 | F | TJ |  |  |  |  |  |  |
|  |  |  |  |  |  |  |  | 50 | M | HN | 58 | M | HN |  |  |  |  |  |  |
|  |  |  |  |  |  |  |  | 50 | F | HN | 58 | F | SC |  |  |  |  |  |  |
|  |  |  |  |  |  |  |  | 50 | F | TJ | 58 | M | SC |  |  |  |  |  |  |
|  |  |  |  |  |  |  |  | 50 | M | HN | 58 | F | TJ |  |  |  |  |  |  |
|  |  |  |  |  |  |  |  | 50 | F | SX | 58 | F | SH |  |  |  |  |  |  |
|  |  |  |  |  |  |  |  | 50 | F | SC | 58 | M | YN |  |  |  |  |  |  |
|  |  |  |  |  |  |  |  | 50 | M | YN | 58 | F | SH |  |  |  |  |  |  |
|  |  |  |  |  |  |  |  | 50 | M | HN | 59 | F | TJ |  |  |  |  |  |  |
|  |  |  |  |  |  |  |  | 50 | F | HN | 59 | M | TJ |  |  |  |  |  |  |
|  |  |  |  |  |  |  |  | 50 | F | SC | 59 | F | SC |  |  |  |  |  |  |
|  |  |  |  |  |  |  |  | 50 | F | SC | 59 | F | HN |  |  |  |  |  |  |
|  |  |  |  |  |  |  |  | 50 | M | SC | 59 | M | SC |  |  |  |  |  |  |
|  |  |  |  |  |  |  |  | 50 | F | SC | 59 | F | HN |  |  |  |  |  |  |
|  |  |  |  |  |  |  |  | 50 | M | SH | 59 | F | TJ |  |  |  |  |  |  |
|  |  |  |  |  |  |  |  | 50 | M | TJ | 59 | M | SC |  |  |  |  |  |  |
|  |  |  |  |  |  |  |  | 50 | F | HN | 59 | M | SC |  |  |  |  |  |  |
|  |  |  |  |  |  |  |  | 50 | F | SC | 60 | F | HN |  |  |  |  |  |  |
|  |  |  |  |  |  |  |  | 50 | F | ZJ | 60 | F | HN |  |  |  |  |  |  |
|  |  |  |  |  |  |  |  | 50 | M | SC | 60 | M | TJ |  |  |  |  |  |  |
|  |  |  |  |  |  |  |  | 50 | M | SC | 60 | F | HN |  |  |  |  |  |  |
|  |  |  |  |  |  |  |  | 50 | M | SX | 60 | F | HN |  |  |  |  |  |  |
|  |  |  |  |  |  |  |  |  |  |  | 60 | F | YN |  |  |  |  |  |  |
|  |  |  |  |  |  |  |  |  |  |  | 60 | F | HN |  |  |  |  |  |  |
|  |  |  |  |  |  |  |  |  |  |  | 60 | M | SC |  |  |  |  |  |  |
|  |  |  |  |  |  |  |  |  |  |  | 60 | F | TJ |  |  |  |  |  |  |
|  |  |  |  |  |  |  |  |  |  |  | 60 | M | SC |  |  |  |  |  |  |
|  |  |  |  |  |  |  |  |  |  |  | 60 | F | HN |  |  |  |  |  |  |
|  |  |  |  |  |  |  |  |  |  |  | 60 | F | HN |  |  |  |  |  |  |

* Abbreviations used in this table: Avrg: Average; Gndr: Gender; Prov: Province; BJ: Beijing; GD: Guangdong; HN: Henan; SH: Shanghai; SX: Shanxi; SC: Sichuan; TJ: Tianjin; YN: Yunnan; ZJ: Zhejiang.

**Supplementary Table 2. Information of recruited cancer patients.**

| Cancer Patients | | | | | | | | |
| --- | --- | --- | --- | --- | --- | --- | --- | --- |
| Total Number | Male | | Female | | Solid Tumor | | Hematological Tumor | Average Age |
| 60 | 25 | | 35 | | 41 | | 19 | 54.1 |
| Detail List | Age | Gender | | Province | | Cancer Type & Detail Information | | |
|  | 80 | M | | Sichuan | | Lung Cancer. PET-CT showed mass at upper branch bronchus of left upper lobe, with increased glucose metabolism in the mass and left hilar lymph nodes. | | |
|  | 56 | M | | Henan | | Lung Cancer. Small cell carcinoma, T4N3M0. | | |
|  | 62 | M | | Henan | | Lung Cancer. On the left lung. | | |
|  | 71 | M | | Henan | | Lung Cancer. Small cell carcinoma, TxN3M1c. | | |
|  | 76 | F | | Henan | | Lung Cancer. Small cell carcinoma, T2bN0M0. | | |
|  | 61 | M | | Henan | | Lung Cancer. Adenocarcinoma, T1cN3M1a. | | |
|  | 71 | M | | Henan | | Lung Cancer. Adenocarcinoma, TxN1M1b. | | |
|  | 54 | M | | Henan | | Lung Cancer. Squamous cell carcinoma, T2N0M0. | | |
|  | 52 | M | | Sichuan | | Lung Cancer | | |
|  | 52 | F | | Henan | | Lung Cancer | | |
|  | 47 | F | | Beijing | | Lung Cancer. Adenocarcinoma in left lung, stage IV, with liver and bone metastases. Under chemotherapy. | | |
|  | 65 | F | | Henan | | Esophageal Carcinoma | | |
|  | 71 | M | | Henan | | Esophageal Carcinoma | | |
|  | 68 | M | | Henan | | Esophageal Carcinoma | | |
|  | 67 | M | | Henan | | Esophageal Carcinoma | | |
|  | 60 | M | | Henan | | Esophageal Carcinoma | | |
|  | 50 | M | | Henan | | Esophageal Carcinoma | | |
|  | 57 | M | | Henan | | Esophageal Carcinoma. Squamous cell carcinoma. | | |
|  | 72 | F | | Sichuan | | Esophageal Carcinoma. Bloodd sample collected 3 years after surgery. | | |
|  | 74 | F | | Sichuan | | Pancreatic Carcinoma | | |
|  | 61 | F | | Sichuan | | Cholangiocarcinoma | | |
|  | 45 | F | | Sichuan | | Liver Cancer. With hepatitis B and cirrhosis. | | |
|  | 39 | M | | Sichuan | | Liver Cancer | | |
|  | 53 | M | | Sichuan | | Liver Cancer. With hepatitis B. | | |
|  | 64 | M | | Sichuan | | Liver Cancer. With hepatitis B. Diagnosed for 3 years before surgery. Blood sample collected 3 days after surgery. | | |
|  | 55 | F | | Sichuan | | Breast Cancer | | |
|  | 47 | F | | Sichuan | | Cervical Carcinoma. Squamous cell carcinoma, IB3. Blood sample collected before the first pre-surgery chemotherapy. | | |
|  | 57 | F | | Sichuan | | Cervical Carcinoma. Poorly differentiated squamous cell carcinoma, IB1. | | |
|  | 56 | F | | Sichuan | | Cervical Carcinoma. Stage III. Blood sample collected before the second pre-surgery chemotherapy. | | |
|  | 43 | F | | Sichuan | | Endometrial Carcinoma. Poorly differentiated endometrioid adenocarcinoma, stage IIIc. Blood sample collected before the first post-surgery chemotherapy. | | |
|  | 45 | F | | Sichuan | | Endometrial Carcinoma. Well differentiated endometrioid adenocarcinoma, stage IIIa. Blood sample collected before the first post-surgery chemotherapy. | | |
|  | 56 | F | | Sichuan | | Endometrial Carcinoma. Well differentiated endometrioid adenocarcinoma, stage Ia. Blood sample collected before the first post-surgery chemotherapy. | | |
|  | 55 | F | | Sichuan | | Endometrial Carcinoma | | |
|  | 52 | F | | Sichuan | | Ovarian Carcinoma. High-grade serous adenocarcinoma on the left ovary, stage Ia. Blood sample collected before the first post-surgery chemotherapy. | | |
|  | 64 | F | | Sichuan | | Ovarian Carcinoma. Poorly differentiated serous adenocarcinoma on the left ovary, stage IIa. The second time post-surgery relapse. Blood sample collected before the third post-relapse chemotherapy. | | |
|  | 56 | F | | Sichuan | | Ovarian Carcinoma. High-grade serous adenocarcinoma on both ovaries, stage IIIc. Blood sample collected before the first post-surgery chemotherapy | | |
|  | 49 | F | | Sichuan | | Ovarian Carcinoma. Clear cell carcinoma, stage IIa. Blood sample collected before the first post-surgery chemotherapy. | | |
|  | 57 | F | | Sichuan | | Ovarian Carcinoma. High-grade serous adenocarcinoma, stage IIIc. Blood sample collected before the third post-surgery chemotherapy. | | |
|  | 53 | F | | Sichuan | | Ovarian Carcinoma. High-grade serous adenocarcinoma, stage IIIc. The second time post-surgery relapse. Blood sample collected before the first post-relapse chemotherapy. | | |
|  | 57 | F | | Sichuan | | Ovarian Carcinoma. Low grade serous adenocarcinoma, stage IIIc. The third time post-surgery relapse. Blood sample collected before the first post-relapse chemotherapy. | | |
|  | 60 | F | | Sichuan | | Ovarian Carcinoma. Stage IIIc. | | |
|  | 53 | M | | Henan | | Malignant Melanoma. On left big toe. | | |
|  | 52 | M | | Henan | | Malignant Melanoma. On left foot. | | |
|  | 57 | F | | Henan | | Malignant Melanoma. On left occiput. | | |
|  | 50 | F | | Henan | | Malignant Melanoma. On left crus. | | |
|  | 47 | F | | Sichuan | | Malignant Melanoma. In vagina. Blood sample collected before the first chemotherapy. | | |
|  | 51 | F | | Sichuan | | Acute Myelogenous Leukemia. Stage M4. Blood sample collected 4 years after bone marrow transplantation. | | |
|  | 22 | F | | Sichuan | | Acute Myelogenous Leukemia. Blood sample collected 1 years after bone marrow transplantation. | | |
|  | 28 | F | | Sichuan | | Acute Myelogenous Leukemia. Blood sample collected 10 months after bone marrow transplantation. | | |
|  | 28 | F | | Sichuan | | Acute Myelogenous Leukemia. Blood sample collected before bone marrow transplantation, and 10 days after the end of radiation and chemotherapy. CR MRD(-). | | |
|  | 55 | F | | Henan | | Diffuse Large B-cell Lymphoma | | |
|  | 83 | M | | Sichuan | | Diffuse Large B-cell Lymphoma | | |
|  | 66 | M | | Beijing | | Diffuse Large B-cell Lymphoma. Primary refractory DLBCL. | | |
|  | 55 | F | | Beijing | | Diffuse Large B-cell Lymphoma. Primary refractory DLBCL. | | |
|  | 44 | M | | Beijing | | Diffuse Large B-cell Lymphoma. Primary refractory DLBCL. | | |
|  | 59 | M | | Beijing | | Diffuse Large B-cell Lymphoma. Primary refractory DLBCL, transformed from follicular lymphoma. | | |
|  | 36 | F | | Sichuan | | B-lineage Acute Lymphoblastic Leukemia. Blood sample collected before bone marrow transplantation, and 10 days after the end of radiation and chemotherapy. | | |
|  | 18 | M | | Sichuan | | B-lineage Acute Lymphoblastic Leukemia. The first bone marrow transplantation on 2018/3/30, then the second transplantation on 2018/8/30 upon suspected relapse. Blood sample collected 13 months after the second transplantation | | |
|  | 41 | F | | Sichuan | | B-lineage Acute Lymphoblastic Leukemia. Blood sample collected 2 months after bone marrow transplantation | | |
|  | 12 | M | | Sichuan | | B-lineage Acute Lymphoblastic Leukemia. Blood sample collected before bone marrow transplantation | | |

**Supplementary Table 3. TRTmix primers**

| Primer Name | Primer Sequence 5’-3’ |
| --- | --- |
| hTCRb-RT1 | TGGGAGAT |
| hTCRb-RT2 | CTTTTGGG |
| hTCRb-RT3 | CCAGTGTG |
| hTCRb-RT4 | CTCTGCTTCTGATGGCTCAAACACAGC |

**Supplementary Table 4. Vβmix primers**

| Primer Name | Primer Sequence 5’-3’ |
| --- | --- |
| TRBV2 | GGTCACACAGATGGGACAGGAAGTGATC |
| TRBV3-1 | GACAAGTCCATTAAATGTGAACAAAATCTGGGCCATGATAC |
| TRBV4-123 | ACACCTGGTCATGGGAATGACAAATAAGAAGTCTTTG |
| TRBV5-1 | ATCTGATCAAAACGAGAGGACAGCAAGTGACA |
| TRBV5-4568 | ACACACCTGATCAAAACGAGAGGACAGCA |
| TRBV6-1235 | GTCCTGAAGACAGGACAGAGCATGAC |
| TRBV6-4 | CAGATCCTGGCAGCAGGACGG |
| TRBV6-6 | CGCATCCTGAAGATAGGACAGAGCATGAC |
| TRBV6-89 | CACATCCTGAAGACAGGACAGAGCATGAC |
| TRBV7-23 | CCCAGTAACAAGGTCACAGAGAAGGGA |
| TRBV7-4 | AGGTACAAAGTCGCAAAGAGGGGACG |
| TRBV7-67 | CCAGGTACAAAGTCACAAAGAGGGGACA |
| TRBV7-8 | GTCGCAAAGAGAGGACAGGATGTAGCT |
| TRBV7-9 | GACACAAGATCACAAAGAGGGGACAGAATGTAACT |
| TRBV9 | GAGATGCTCCCCTAGGTCTGGAGAC |
| TRBV10-12 | GATCACAGAGACAGGAAGGCAGGTGAC |
| TRBV10-3 | GGTCACAGAGACAGGAACACCAGTGAC |
| TRBV11-1 | GGTGTGATCCTATTTCTGGCCATGCTACC |
| TRBV11-2 | GGTGCAATCCTATATCTGGCCATGCTACC |
| TRBV11-3 | GGTGCAATCCTATTTCTGGCCACAATACC |
| TRBV12-34 | AGGTGACAGAGATGGGACAAGAAGTGACT |
| TRBV12-5 | GCACAAGGTGACAGAGATGGGACAAGAAG |
| TRBV13 | CTGAAATGCTATCCTATCCCTAGACACGACACTG |
| TRBV14 | GCGTAATAGAGAAGGGCCAGACTGTGAC |
| TRBV15 | GCCAGTGACCCTGAGTTGTTCTCAGAC |
| TRBV16 | TGTCAGAGGGGAAGGACAGAAAGCA |
| TRBV18 | CTGAGATGCAGCCCAATGAAAGGACAC |
| TRBV19 | CTGAGTTGTGAACAGAATTTGAACCACGATGCC |
| TRBV20-1 | CCTCTGTGAAGATCGAGTGCCGTTCC |
| TRBV24-1 | GCTGGAATGTTCTCAGACTAAGGGTCATGATAGAATGTAC |
| TRBV25-1 | GGAATGTTCTCAAACCATGGGCCATGAC |
| TRBV27 | CACAGTGACTGGAAAGAAGTTAACAAGTGACTTGTTCTCA |
| TRBV28 | TCTGGAATGTGTCCAGGATATGGACCATGA |
| TRBV29-1 | CCAGTGTCAAGTCGATAGCCAAGTCACC |
| TRBV30 | GGAGTGCACTGTGGAGGGAACATCA |

**Supplementary Table 5. hTCRCbBCx primers**

| Primer Name | Primer Sequence 5’-3’ |
| --- | --- |
| hTCRCbBC1 | gagTTACTCGCgcacagcgacctcgggtgggaac |
| hTCRCbBC2 | gagTCGTTAGCgcacagcgacctcgggtgggaac |
| hTCRCbBC3 | gagTACCGAGCgcacagcgacctcgggtgggaac |
| hTCRCbBC4 | gagTGTTCTCCgcacagcgacctcgggtgggaac |
| hTCRCbBC5 | gagTTCGCACCgcacagcgacctcgggtgggaac |
| hTCRCbBC6 | gagTTGCGTACgcacagcgacctcgggtgggaac |
| hTCRCbBC7 | gagTCTACGACgcacagcgacctcgggtgggaac |
| hTCRCbBC8 | gagTGACAGACgcacagcgacctcgggtgggaac |
| hTCRCbBC9 | gagTAGAACACgcacagcgacctcgggtgggaac |
| hTCRCbBC10 | gagTCATCCTAgcacagcgacctcgggtgggaac |
